# The Aryl Hydrocarbon Receptor and the Nervous System

**DOI:** 10.3390/ijms19092504

**Published:** 2018-08-24

**Authors:** Ludmila Juricek, Xavier Coumoul

**Affiliations:** 1 Toxicologie Pharmacologie et Signalisation Cellulaire, INSERM UMR-S 1124, 45 rue des Saints-Pères, 75006 Paris, France; ludmila.juricek@outlook.com; 2 UFR des Sciences Fondamentales et Biomédicales, Université Paris Descartes, Sorbonne Paris Cité, 45 rue des Saints-Pères, 75006 Paris, France

**Keywords:** AhR, nervous system, endocrine disruptor, TCDD, dioxin, neuron

## Abstract

The aryl hydrocarbon receptor (or AhR) is a cytoplasmic receptor of pollutants. It translocates into the nucleus upon binding to its ligands, and forms a heterodimer with ARNT (AhR nuclear translocator). The heterodimer is a transcription factor, which regulates the transcription of xenobiotic metabolizing enzymes. Expressed in many cells in vertebrates, it is mostly present in neuronal cell types in invertebrates, where it regulates dendritic morphology or feeding behavior. Surprisingly, few investigations have been conducted to unravel the function of the AhR in the central or peripheral nervous systems of vertebrates. In this review, we will present how the AhR regulates neural functions in both invertebrates and vertebrates as deduced mainly from the effects of xenobiotics. We will introduce some of the molecular mechanisms triggered by the well-known AhR ligand, 2,3,7,8-tetrachlorodibenzo-p-dioxin (TCDD), which impact on neuronal proliferation, differentiation, and survival. Finally, we will point out the common features found in mice that are exposed to pollutants, and in AhR knockout mice.

## 1. Introduction

The aryl hydrocarbon receptor (AhR) is a protein that belongs to the PAS (Per-ARNT-Sim) family and contains a basic helix-loop-helix domain. The AhR is present in the cytoplasm of many vertebrate cell types as part of a complex that is composed of a dimer of the chaperone heat shock protein (HSP) 90, an immunophilin-like protein called X-associated protein (XAP) 2 or AIP1, the phosphoprotein p23, and the non-receptor protein tyrosine kinase also known as pp60^src^ [1].

Initially, the AhR was characterized as a xenobiotic receptor, and many different types of xenobiotics are known to be ligands for the AhR [2]. Binding to a ligand presumably induces a conformational change in the AhR and leads to dissociation of the complex, which translocates into the nucleus where it interacts with ARNT (aryl hydrocarbon nuclear translocator or hypoxia-inducible factor-1ß), to forms a transcription factor. This heterodimer binds to xenobiotic-responsive elements (XRE) in the promoters of target genes, and regulates their transcription in part, to control xenobiotic metabolism.

During the previous decade, several additional functions for this protein have been discovered. First, the discovery of endogenous ligands, which include indole derivatives, or kynurenin in the brain, or tryptophan metabolites, suggested that the AhR might function in normal physiology [3]. Second, xenobiotics that bind to the AhR have been used as disruptors to help identify the physiological functions of the receptor [4]. All of this, combined with the use of animal knockout models, has demonstrated that the AhR is involved in the regulation of several physiological processes, including intestinal homeostasis, development, behavior, and immunological responses [3].

In the invertebrate nematode model, *Caenorhabditis elegans*, AHR-1 displays some interesting features [5,6,7,8]. First, it does not bind 2,3,7,8-tetrachlorodibenzo-p-dioxin (TCDD), the prototypical ligand for the AhR of vertebrates. Second, it is expressed mostly in neuronal cell types such as GABAergic neurons. Although a role for the AhR in modulating behavior or in the functioning of the hypothalamic-pituitary axis has been surmised, there have been few investigations on the function of the AhR in the nervous systems of vertebrates, despite the fact that it regulates feeding behavior or dendritic morphology in invertebrates [9,10,11]. In this review, we will present some of the interesting features regarding the role of the AhR in the regulation of neural functions in both invertebrates and vertebrates, as deduced mainly from the effects of xenobiotics. In addition, we will point out the common features found in mice exposed to pollutants and in AhR knockout mice.

## 2. Expression and Functions of the AhR in Invertebrates

In 2006, Kim et al. showed that the AhR ortholog in *Drosophila*, named Spineless (Ss), was involved in the morphogenesis of dendrites: Ss deficiency leads to a decreased number of dendrites in highly branched sensory neurons, whereas it increases the number of dendrites in low-branched sensory neurons. Ss is probably not the only protein that is involved in the differentiation of both types of sensory neurons, because its level of expression is similar in both of the cell types. Tango (the ortholog of ARNT), however, is not involved in this differentiation process, suggesting that the formation of the heterodimer Ss-Tango is not required [12].

Spineless also has been implicated in the formation of the eye unit (ommatidium) [13]. Each eye is composed of 800 units. Each unit is composed of eight photoreceptors (R1–R8). R1–R6 act as rods and R7 and R8 function as cones. The level of expression of Ss controls the phenotypic fate of R7, which can be classified as yellow or pale. Ss deficiency leads to the loss of yellow ommatidia. In 2011, we showed [14] that the human AhR controls the expression of Sos1 (Son of Sevenless), another gene that was initially identified in the phenotypic specification of R7 in *Drosophila*. It would be interesting to determine whether Ss controls the expression of Sos1 in *Drosophila*, to elucidate the pathway by which the R7 phenotype is established.

In *Caenorhabditis elegans*, the AhR ortholog, AHR-1, is also involved in neuronal differentiation. Huang et al. showed that two types of GABAergic motoneurons, which control the head movements, express AHR-1. Deficiency of AHR-1 slightly modifies the phenotype of these motoneurons towards another type that regulates the head movements in another direction [5]. This differentiation also depends on AHA-1, the ortholog of ARNT, but strangely not on DAF21, the ortholog of Hsp90. AHR-1 is also important for the differentiation of neurons controlling social feeding behavior, and specifically in the regulation of the expression of two soluble guanylyl cyclases that are essential for the regulation of this behavior [6,7]. Finally, AHR-1 and AHA-1 might be involved in the transcriptional regulation of a transmembrane receptor and tyrosine kinase, CAM1, which, as a Wnt antagonist, assists in the formation of gap junctions between one type of interneurons (BDU) and one type of mechanoreceptor (PLM) [8]. This mechanoreceptor is important for detecting touch stimuli in the posterior part of the nematode, while the BDU interneuron likely transduces signals from the PLM, to promote forward locomotion.

The patterns of expression of Spineless and AHR-1 (mostly localized in neurons in invertebrates), and the phenotypes associated with their deficiencies, strongly suggest that the function of these two orthologs is critical for differentiation and the establishment of neuronal fate. No ligand has been characterized for either of these proteins, even though their signaling pathways have features in common with those characterized in vertebrates (such as ARNT as a partner, or XREs located in the promoters of target genes, among others) [15]. One hypothesis is that during evolution, a ‘xenobiotic receptor’ function was acquired due to mutations in the PAS domain. This hypothesis raises the question as to whether and how these mutations have affected the ancestral functions of AhR orthologs in neurons and neural cells in present-day vertebrates.

## 3. Expression of the AhR in the Nervous System of Vertebrates

**Figure 1:**
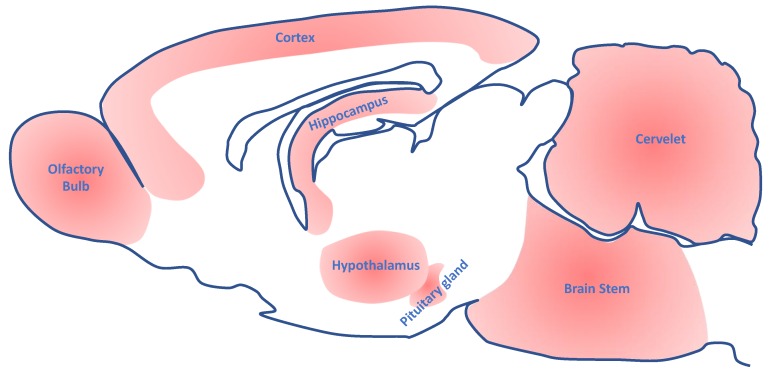
Representation of the rat brain domains that express the aryl hydrocarbon Receptor.

The expression of the AhR has been characterized in several animal models using immunohistochemistry and hybridization in situ [16], with a recent study by Kimura and colleagues, who showed specific temporal and spatial patterns of the AhR messenger RNA (mRNA) expression in the mouse cortex, hippocampus, cerebellum, olfactory bulb, and rostral migratory stream [17]. The expression of the AhR was also studied in other animal models. Kainu et al. showed that, in the rat brain, the AhR mRNA is expressed mainly in the neurons of the cortex, the cerebellum, the hippocampus, and the olfactory bulb (together with ARNT mRNA) (Figure 1). The widespread distribution in the brain of AhR and ARNT was confirmed by other methodologies such as real-time quantitative polymerase chain reaction (PCR) in mice. The brainstem and several nuclei of the hypothalamus (including the suprachiasmatic nucleus, which controls circadian rhythmicity, see below) have markedly higher AhR amounts than the other regions of the brain. In vivo and in vitro data also suggest that the pituitary gland expresses the AhR [18].

In light of the role of AhR orthologs in neuronal differentiation in invertebrates, it is of interest to understand how the AhR is developmentally regulated in vertebrates. In neural cells, the AhR is expressed in the early developmental stages: the mRNA is detected in the neural progenitor cells in the mouse hippocampus [19,20] and its expression increases throughout development. Similarly, it is found in immature murine cerebellar granule cells [21,22]. We demonstrated that the receptor is present in retinal ganglion cells (RGC) of murine embryos, depending on the developmental stage [23]. Gohlke et al., also suggested that the AhR is expressed in the murine telencephalon (E13.5), probably in GABAergic neurons [24]. Astrocytes and endothelial cells isolated from the murine blood brain barrier also express the AhR protein, thus demonstrating that its expression is not restricted only to neuronal progenitors or neurons [25,26]. Pravettoni et al. also demonstrated that the protein is expressed also in glial cells [19], regulating neuron survival [27].

As has been shown in the liver, the expression of the AhR in the brain also exhibits chrono-periodicity. Periodic variations in the level of the AhR mRNA were observed in the murine suprachiasmatic nucleus, a small region of the hypothalamus that controls circadian rhythmicity [28,29]. As a consequence, the mRNA level of cytochrome P450 1A1, which is used as a biomarker of TCDD exposure, also cycles in this region.

Finally, regulation of the expression of the AhR in the nervous system depends not only on internal stimuli, but also on traumatic brain injury (TBI) [30], exposure to other xenobiotics, and endocrine disruptors such as bisphenol A (BPA) or di-n-butyl phtalalte [31]. BPA binds and activates different nuclear receptors, and it increases the level of the AhR transcript in the cerebellum of both female and male murine embryos [32]. In addition, AhR ligands, such as the ones found in Aroclor 1254, also increase the expression of the AhR in the mouse hypothalamus. The expression of ARNT also is regulated by environmental factors. Kitamura et al. demonstrated that ARNT mRNA is increased in the rat hippocampus upon exposure to kainic acid (found in some Asian foods), which is an agonist for the ionotropic glutamate receptor. However the levels of the AhR transcript remain unchanged [33].

The presence and the regulation of the expression of the AhR throughout the brain has led to the study of the effects of AhR ligands, and in particular TCDD, on the nervous system in vertebrates.

## 4. Disruption of Neuroendocrine Functions by AhR Ligands

Over 35 years ago, Elovaara et al. first investigated the effects of TCDD on the nervous system in rats [34]. Later, it was shown that the brains of birds were affected (asymmetry) by intoxication with TCDD [35,36]. Neuroendocrine functions were among the first type of functions studied following such intoxication (later, the expression of the AhR was coherently detected in structures involved in neuroendocrine functions, including the hypothalamus and the pituitary gland [28,37]).

In the rainbow trout, Aluru et al. characterized the whole brain transcriptome following exposure to AhR ligands. ß-naphtoflavone modulated the mRNA levels of 49 genes involved in neuroendocrine functions (stress and reproduction); the authors demonstrated that resveratrol can partly antagonize BNF for only 27% of the genes [38]. BNF can be considered to be a specific ligand of the AhR. However, resveratrol has a broad spectrum of action, including effects on the AhR. Thus, caution should be used when interpreting the effects of resveratrol when it is assumed that the compound is a pure antagonist [39].

**Figure 2:**
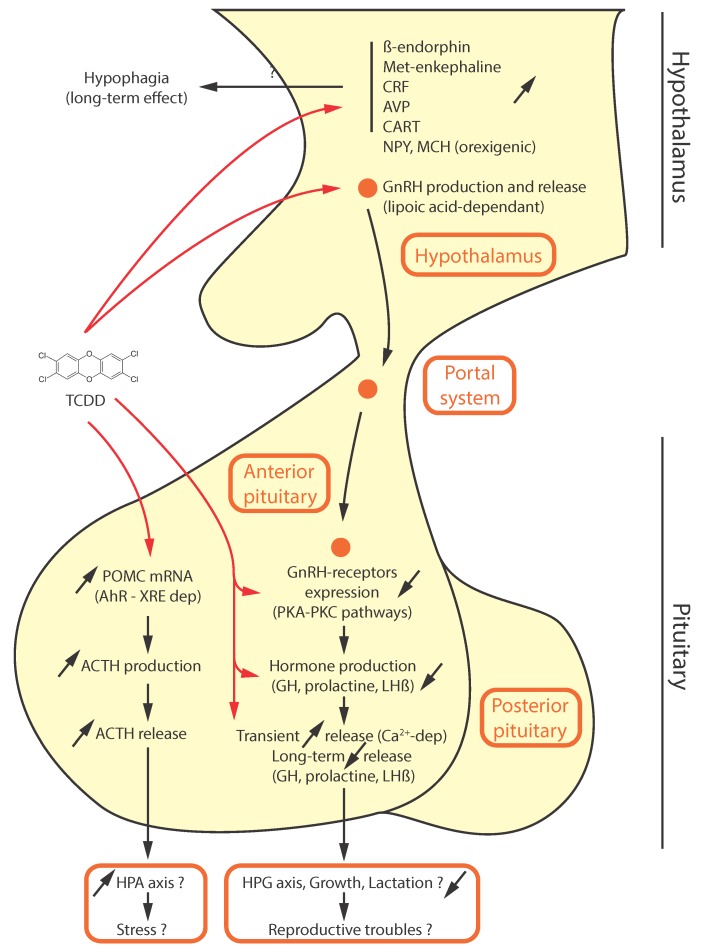
The putative effects of tetrachlorodibenzo-p-dioxin (TCDD) on the hypothalamic-pituitary axis. CRF: corticotropin-releasing factor; AVP: arginine vasopressin; CART: cocaine- and amphetamine-regulated transcript; NPY: neuropeptide Y; MCH: melanin-concentrating hormone; GnRH: gonadotropin-releasing hormone; PKA: protein kinase A; PKC: protein kinase C; GH: growth hormone; Ca^2+^: calcium ion; LHß: luteinizing hormone beta; POMC: proopiomelanocortin; XRE: xenobiotic responsive element; ACTH: Adrenocorticotropic hormone; HPA: hypothalamic-pituitary-adrenal; HPG: hypothalamic-pituitary-gonadal. Red arrow: Molecular Initiating Event; Black arrow: Key Event; Red dot–Black Text: Adverse Outcome; Red dot–Red Text: Organ or system; Red circle: GnRH.

Most of the studies of neuroendocrine disruption and AhR ligands have been conducted in rodents (Figure 2). Exposure to a mixture of AhR ligands that are present in breast milk by gavage to young rats (postnatal days 1 and 20) led to a reduction of estrogen receptor (ER) mRNA levels in the whole brain. In contrast, the expression was increased for two enzymes in the cortex which sequentially inactivate estrogens: cytochrome P450 1b1 (Cyp1b1) and membrane-bound catechol-o-methyltransferase (Comt) [40]. This might be important in relation to the neuroprotective effects of estrogens. The results suggest that the AhR disrupts estrogen signaling in the brain by decreasing both ER and estrogen contents. This is concordant with the observation that TCDD specifically decreased the sensitivity of the hypothalamus to circulating hormones such as estradiol (E2) [41]. This hyposensitivity could be due to a decreased DNA-binding activity of the receptor (possibly due to lower ER levels) or to lower plasma levels of estrogen [42]. This decreased sensitivity was also observed for the pituitary gland (see below). The effects of TCDD on both the hypothalamus and the pituitary gland have been studied both in vitro and in vivo. Exposure of rats to high doses of TCDD reduced food intake, influenced body composition [43], and increased the expression of ß-endorphin (which might be associated with hypophagia) [44] and Met-enkephalin (which regulates pain as well as food intake and body temperature) in the hypothalamus [45]. The expression of corticotropin-releasing factor (CRF) and vasopressin (AVP) in the paraventricular nucleus (PVN) was also altered by high doses of TCDD in Sprague-Dawley (SD) rats, a phenomenon associated with reduced water and food intake [46]. A significant increase of CRF in the hypothalamus was also observed in monkeys (daily low oral doses of TCDD), associated with an increase of cortisol in the blood [47]. Fetissov et al. also showed that neuropeptide Y (NPY), cocaine, and amphetamine-regulated transcript (CART) and melanin-concentrating hormone (MCH) mRNA were increased by TCDD (single oral administration) in adult male SD rats [48]. Moreover, in the rat GnV-3 hypothalamic cell line, TCDD (10 nM, 6 h) increased mRNA expression of NPY [49]. While increased expression of NPY or MCH (orexigenic factors) might be a paradox regarding the loss of appetite described following TCDD exposure, the regulation of those factors might be a little more complex in time; indeed, a biphasic effect of TCDD on orexigenic factors (an initial suppression followed by an increase in expression) has also been described [50]. More recently, a study of the effect of TCDD (1 µg/kg at GD15) on the fetal rat hypothalamus metabolome (at GD20) showed significant decreases of several amino acids (alanine, aspartate, glutamine, isoleucine, phenylalanine, and threonine) which could impact the levels of some neurotransmitters [51].

TCDD and the AhR also influence the hypothalamic-pituitary-gonadal axis (HPG axis). Indeed, low doses of TCDD (single dose at GD15 by gavage) accelerates puberty and maturation of the HPG axis in female Long-Evans (LE) rats [52]. As previously evoked, the antagonism between the AhR and the ER signaling pathways has a major effect on the highly E2-sensitive pituitary gland. Crosstalk has been reported between the AhR and the ERα both in lactotropes (prolactin-secreting cells), and in gonadotropes (FSH(Follicle Stimulating Hormone)- and LH(luteinizing hormone)-secreting cells). For example, TCDD blocked E2-induced prolactin expression, but induced LH-ß and ERα mRNA expression [37]. This effect on prolactin expression was also demonstrated in other models [18,53]. Moran et al. demonstrated that TCDD decreased prolactin release in the GH3 rat somatolactotrope tumor cell line. This might be due to an increased secretion of dopamine by the hypothalamus following stimulation by the pollutant, since dopamine impairs prolactin secretion [53]. The exposure to TCDD (1 µg/kg, gavage, GD15) also inhibited the levels of FSH mRNA in the pituitary [54]. However, the plasma gonadotropin levels were not affected [37,54]. Nevertheless, these results may depend on the time course of the experiment and the hormonal status of the model. For example, Li et al. demonstrated a transient release of gonadotropins, following a 24 h exposure to TCDD (0.03–30 µg/kg, gastric intubation), by a calcium-dependent, GnRH(gonadotropin-releasing hormone)-independent mechanism [55]. The role of calcium on the effects of TCDD may be important in several regions of the brain, and this will be discussed later.

The effects of exposure to TCDD seem to depend on the developmental stage, since there is a decreased production (but not secretion) of these gonadotropins, following exposure (1 μg/kg, gavage, GD15) in the rat fetus [51,56,57,58], but not the adult [59]; Bookstaff et al. previously demonstrated that TCDD decreased the number of GnRH receptors in the pituitary gland, which suggests that the sensibility of this region to hypothalamic GnRH is decreased [60,61]. Moreover, GnRH production and release is also inhibited by TCDD (5 µg/kg, GD15, gavage) in male rats, due to ultra-structural alterations of the GnRH-positive neurons [62,63]. However, this point is controversial in light of the results of Trewin et al., which do not show any alterations with short-term hormone release from adult female rat hypothalamic and pituitary explants, following exposure to 3.1 nM TCDD in the perfusion medium [64]. In terms of signaling, it has been hypothesized that GnRH can stimulate the activity of two protein kinases A and C (PKA and PKC), to increase gonadotropin expression, and that those two pathways are then be targeted by TCDD [65]. Moreover, the inhibition of GnRH release by TCDD can be relieved by co-treatment with lipoic acid, a cofactor of several mitochondrial enzymes. TCDD has been shown to alter the metabolism of the hypothalamus and the pituitary gland, in particular by decreasing the content of lipoic acid. Due to the function of lipoic acid as a co-factor in the pyruvate dehydrogenase and α-ketoglutarate dehydrogenase complexes, the decrease in lipoic acid decreases the level of adenosine triphosphate (ATP), and subsequently decreases the secretion of the gonadotropins [58].

In addition to the effects of TCDD being dependent on the developmental stage and circadian rhythmicity of an organism, they also appear to depend on the dose of TCDD and the chronology of exposure. Exposure of isolated pituitary glands to low doses of TCDD (<0.1 nM) might decrease the levels of GH and prolactin mRNA in isolated pituitary glands, whereas the opposite effect was observed at higher doses (>1 nM) [66]. One hypothesis is that TCDD acts positively on the release of gonadotropins in a calcium-dependent manner in the short term, but that it shuts down the HPG axis and impacts on rodent reproduction in the long term. In mice, the effects of exposure to TCDD on the expression and secretion by the pituitary gland of other peptides also has been studied [67]: TCDD (high single oral doses) increased the level of pro-opiomelanocortin (POMC) mRNA. POMC is a precursor of many small peptides with diverse functions. As a consequence, TCDD increased the levels of both adrenocorticotrophic hormone (ACTH) and ß-endorphin [48]. ACTH activates the release of glucocorticoids by the adrenal gland, whereas ß-endorphin acts as an analgesic peptide. Putative XREs are present in the promoter of the POMC gene. POMC could thus be a TCDD-activated AhR target gene; the increased production of POMC mRNA has been observed in other models: mice [68] and rat models, including primary anterior rat cell cultures [68,69], the AtT-20 cell line [48,67], and Sprague-Dawley rats [46]. However, the mechanism for the effects of TCDD may be more complex since TCDD (<10 nM) increases ACTH production and secretion, but also decreases CRF-stimulated ACTH secretion in rat primary anterior cell cultures [68].

Thyroid hormones also depend on the HPG regulatory axis, and TCDD influences the levels of TSH (thyroid stimulated hormone) secreted by the pituitary gland. The levels of thyroid hormones (T4 and T3) and growth hormone (GH) are decreased in rats exposed to TCDD during gestation and lactation (0.2 or 0.4 μg/kg body weight, gavage from GD1 to LD30) [70]. However, this is a controversial issue, since other authors have shown that although T4 is decreased, T3 is not decreased, whereas TSH is increased [71]. This decrease might be also be indirectly due to the induction of the conjugating phase II enzyme, UDP-glucuronosyltransferase (UGT1), which converts thyroid hormones to conjugated and inactive metabolites, but this does not explain the different consequences on the T4 and T3 levels. Conversion of active hormones to conjugated metabolites is highly active in the peripheral organs (liver) also might be responsible for the decreased serum levels of melatonin.

Studies of the impact of TCDD on the function of the HPG axis in rodents have led to the elucidation of several mechanisms that are activated or inhibited by this pollutant. More recently, new light has been brought to bear on other potential pathways that have been altered by the AhR.

## 5. Impact of AhR Ligands on Neurogenesis, Cell Proliferation, Differentiation, and Survival in the Nervous System of Vertebrates and Possible Mechanisms

### 5.1. Neurogenesis, Cell Proliferation, Differentiation and Migration

**Figure 3:**
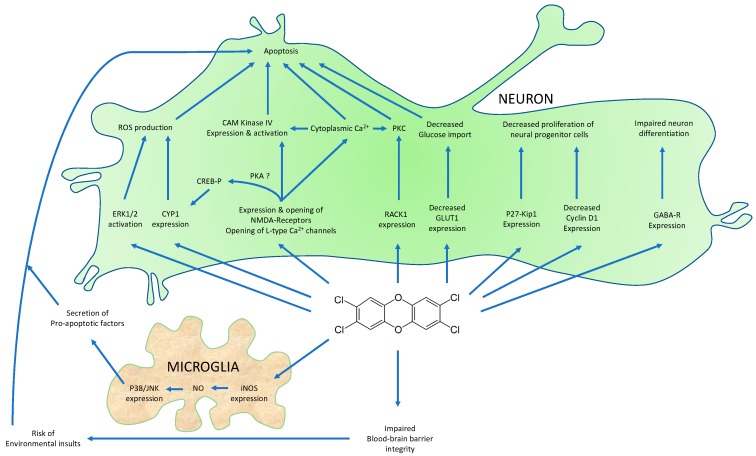
TCDD regulates neuronal cell proliferation, differentiation, and survival. ERK1/2: extracellular-regulated kinase 1/2; CYP1: cytochrome P450 1; CREB: cyclic adenosine monophosphate (cAMP)-responsive element binding protein; PKA: protein kinase A; NMDA: *N*-methyl-d-aspartate; RACK1: Receptor for activated C kinase 1; GLUT1: glucose transporter 1; GABA-R: gamma-aminobutyric acid receptor; iNOS: inducible NO synthase; NO: nitric oxide; JNK: jun-N terminal kinase.

The AhR mRNA is detected in neural progenitor cells in the hippocampus [20], and in early brain structures [24] in mice. Although TCDD (1 nM) does not modify the viability of a neural murine precursor cell line (C17.2), it alters proliferation by blocking the G1/S checkpoint, due to altered levels of expression of p27 Kip1 (increased) and cyclin D1 (decreased) (Figure 3). These effects were confirmed with SK-N-SH human neuronal cells (increased p27) and primary neural precursor cells from the embryonic mouse forebrain [72,73]. TCDD also impairs the neurosphere proliferation of isolated neural precursor cells [74], and the differentiation program in the hippocampus [75], the cortex [76], and the cerebellum. The expression of GABA receptor α6 is decreased in both wild-type mice exposed to TCDD (1 µg/kg, gavage, PND6) and AhR knock-out (−/−) mice, which suggests that TCDD acts a physiological disruptor and an antagonist of endogenous ligands (Figure 3) [22]. However, in vitro experiments with granule neuron precursors showed an increased expression of this GABA receptor following exposure to TCDD, which suggests that the regulation of this marker is possibly time-dependent [22]. TCDD also decreases the expression and the activity of the acetylcholinesterase (AChE) in SK-N-SH human-derived neuronal cells, an enzyme involved in the synaptic recycling of the neurotransmitter, acetylcholine. This effect is predictive to be human-specific as no XRE is detected in the mouse or rat AChE promoters, compared to the corresponding human gene [77].

Beside the neuronal cell type, treatment of C6 glioma with an AhR ligand (ß-naphtoflavone or TCDD) inhibits their differentiation in astrocytes, as suggested by decreased expression of GFAP and interleukin 6, probably due to altered function of STAT2 signaling [78] or favors astrocyte senescence via the activation of the WNT/ß-catenin pathway [79]. Moreover, TCDD could potentially stimulate microglia proliferation through activation of the Akt/GSK-3ß/cyclin D1 pathway [80]. Alteration in the number of astrocytes and microglial cells might have major impacts on the function of neuronal circuits. Indeed, treatment with TCDD of rat hippocampal primary culture, which contains both neurons and astrocytes, disrupts communication between astrocytes and neurons by targeting the gap junctions [81]. Inflammation is well known to activate microglia and astrocytes, a process that is potentially triggered by the AhR; indeed, in the cerebral cortex in fetal mice exposed to TCDD (5 µg/kg body weight, gavage, GD12.5), the levels of two inflammatory chemokines, Cxcl4 and Cxcl7 (markers of rheumatoid arthritis) are increased [82]. The authors highlighted the importance of testing other sites in order to better elucidate the role of the AhR [20]. On this line, the effects of alternative AhR ligands are particularly interesting, as they can display opposite effects. Indeed, indoxyl sulfate, which is a tryptophan metabolite, has been shown to stimulate the AhR in astrocytes, together with neuroinflammation in several cellular astrocyte models [83]. On the contrary, the team of F. Quintana demonstrates the importance of endogenous ligands produced by the microbiota and derived from tryptophan, in the control of inflammatory processes triggered by astrocytes. Such ligands reduce CNS inflammation together with type I interferons [84,85,86].

This is also true with altered glial cells: indeed, several studies have recently underlined the role of the AhR in tumor cell proliferation in the nervous system. Glioblastomas are one of the most aggressive form of tumors, whose proliferation depends on several signaling pathways. In 2011, the laboratory of M. Platten identified kynurenine (KYN), a metabolite from tryptophan, as an AhR ligand that contributes to (1) brain tumor proliferation and (2) abolishment of antitumor immune defenses acting on both tumor and immune cells [87]. More recently, a tryptic AhR-transforming growth factor-ß (TGF-ß)-integrin has been a suspected to play a significant role in glioblastoma malignancy, as the AhR upregulates the expression of several members of the TGF-ß pathway in malignant glioma cells [88]. This appears to be even more complex and linked to adhesion properties of such tumor cells, as both pathways are controlled by integrin signaling [89].

Migration processes of normal neuronal cells might also be disrupted by the receptor [90], as the use of constitutively activated AhR demonstrates that excessive AhR activity impairs neuronal migration in the hippocampus [91]. However, it is also important to mention that species-specific differences may also exist due to differences of AhR affinities or expression [92].

Altogether, those studies show the AhR controls cell proliferation of progenitor cells, but also tumor cells and might represent a future therapeutic target for a large pathological spectrum from neurodegenerative diseases to brain cancers. Finally, cell proliferation/differentiation could not be dissociated from cell death, as a recent study showed that the expression of the AhR battery is strongly impacted over neuronal differentiation [93], with a potential impact on the effects of such ligands on cell death [94].

### 5.2. Cell Survival

The activation of apoptotic signals by AhR ligands has been characterized in several in vitro and in vivo models: mouse primary neuronal cells, mouse cerebellar granule cells, acutely TCDD-exposed female rats, in utero TCDD-exposed male rats, zebrafish larvae [95,96,97,98,99,100]. The results may provide important insights for understanding neurodegeneration, as AhR modulators could represent an interesting approach to protecting neurons from injuries [101]. For example, the expression of both pro- and anti-apoptotic genes is modified in the brains of the offspring of rats exposed to TCDD (gavage at CD15) [102], and cell viability is significantly reduced in rat cortical cells following high levels of exposure [103]. In zebrafish embryos, TCDD stimulates cell death in the dorsal midbrain in an AhR-dependent manner [104]. Finally, in the rat pituitary AtT-20 cell line, low doses of TCDD (<1 nM) also stimulate apoptosis and necrosis [105]. Due to this large number of studies, some authors are able to propose mechanisms of TCDD action (Figure 3):

**Mechanisms of action:** Significant roles in the neurotoxicity of AhR ligands are suspected to be due to the non-genomic pathways that are activated following binding of the ligand to the AhR [106,107,108] including NMDA (*N*-Methyl-d-Aspartate) excitotoxicity, together with an cytoplasmic increase in calcium, generation of reactive oxygen species (ROS), and induction of p27-Kip1 (see above) [109]. In rat neuronal hippocampal cells, TCDD triggers a non-genomic pathway, which results in a rapid increase in cytoplasmic Ca^2+^ within 30 s. This leads to the stimulation of PKC and an alteration of the mitochondrial membrane potential [107]. Activation of PKC by TCDD is observed also in developing neuronal cells, and is associated with an increased level of the RACK-1 protein (receptor of activated C kinase-1) which allows a translocation of PKC to the cell membrane in a AhR-dependent manner [110]. The activation of PKC (and increased RACK-1 expression) by TCDD is observed commonly, for example, in cerebellar granule cells [111], and then could be suspected, in immature cerebellar granule cells, in which AhR ligands decrease cell survival [21]. An effect of TCDD on calcium homeostasis is also suggested by the results of Hong et al., who showed that some excitatory postsynaptic potentials in the rat hippocampus are inhibited by this pollutant, probably through the opening of L-type calcium channels [112].

Concomitantly with the increased levels of cytoplasmic Ca^2+^ and the activation of PKC, activation of ERK-1/2 and the production of ROS has been observed in cerebellar granule cells or in differentiated PC12 cells [113,114]. The inhibition of ERK-1/2 counteracted the effect of TCDD on ROS production. ROS production could be associated with oxidative stress, which is observed at low dose-chronic exposure to AhR ligands. In rats exposed orally to low doses of TCDD and PCB126, markers of oxidative stress were increased in the brain [115]. The level of ROS were higher in the liver tissues as compared to the brain, which led the authors to hypothesize that the blood-brain barrier plays a significant protective role [116]. The same group demonstrated that chronic exposure of mice to low doses of TCDD led to an increased production of superoxide anion, lipid peroxidation, and DNA-strand breaks [117]. A non-monotonous relationship between oxidative stress and the exposure to TCDD exposure was demonstrated in the cerebral cortex and in the hippocampus [118].

As stated above, an increase in cytoplasmic calcium, is also associated with NMDA excitotoxicity. AhR ligands influence glutamate-mediated excitotoxicity (Lin et al. showed that TCDD increases NMDA excitotoxicity, along with an increase in cytosolic Ca^2+^ [119] and activation of caspase 3 [120]), but beside this process, may impact more generally the expression of several neurotransmitter receptors; indeed, exposure of LE rats to TCDD during gestation, decreases the expression of two glutamate receptors including NR2B (NMDA-Receptor 2B) and GluR1 (AMPA receptor), which is associated with a decreased spontaneous activity of cortical neurons [121]. Kakeyama et al. demonstrated that NR2A mRNA is increased as compared to controls in the neo-cortex and the hippocampus at post-natal day 49 (PND49), following in utero exposure to TCDD [122]. In contrast, the levels of NR1 mRNA, and protein in the hippocampus of LE rats are down-regulated by gestational exposure to TCDD during the first postnatal month [123].

Interestingly, NMDA receptors (NR) also appear to influence the activity of the AhR. Cells treated with inhibitors of NMDA receptors or cells that express hypomorphic NMDA-R exhibit decreased expression of AhR target genes. NRs have been shown to trigger (1) the phosphorylation of the CREB (cAMP-responsive element binding protein) protein which associates with the AhR on its target promoters (including that of Cyp1A1), and (2) the activity of the Ca^2+^/calmodulin-dependent protein kinase IV (CAMK-IV), which can modulate the AhR function [124]. CAMK-IV is also stimulated by the AhR and TCDD, which suggests that a regulatory loop might modulate the toxicity of AhR ligands [125].

The genomic pathway activated by the AhR also might be involved in the alterations of proliferation and cell survival. As previously noted, the level of p27-Kip1 (a cyclin-dependent kinase (CDK) inhibitor) is increased by AhR ligands. In addition to its effects on cell proliferation, p27-Kip1 could have a significant effect on apoptotic signaling. In fact, Xu et al., similarly to Latchney et al. [72,120], demonstrated that apoptosis mainly occurs in neurons in which p27 is induced. An increased level of p27 is found also in the cortex of mice that are exposed in utero to TCDD. Consequently, there is a reduction of the cortical cell number, and thinner deep neocortical layers [126]. The mechanism responsible for the induction of p27 has been associated with the activation of the forkhead box class O 3a (FoxO3a) by the AhR and TCDD. The pro-apoptotic effect of AhR ligands via FoxO3a could be enhanced by co-treatment with estrogen receptor-antagonists (ICI 182,780). This could represent another endocrine-disrupting effect via a crosstalk between the ERs and the AhR [127,128]. Beside p27-Kip1 and FoxO3a, the induction of neuronal nitric oxide (NO) synthase identified in PC12 cells upon TCDD exposure represents another cell death-related mechanism [129]. Indeed, high levels of NO are suspected to activate the mitochondrial apoptotic pathway. Finally, the AhR has been identified as a regulator of TDP-43 expression, a biomarker of the development of amyotrophic lateral sclerosis (ALS), suggesting for the first time that a potential link exists between environmental ligands and the development of these neurodegenerative diseases [130].

### 5.3. Indirect Effects of AhR Ligands on Neuron Survival

The influence of AhR ligands on apoptosis could be due also to indirect effects such as the loss of blood brain barrier integrity [104], alteration of essential lipid levels [18,131], disruption of glucose transport (due to a decreased expression of GLUT1 protein) [132], or increased local inflammation, for example from cigarette smoke, which contains AhR ligands such as benzo(a)pyrene [133].

Microglia and macroglia also may have an important role in neuron survival. It has been shown that primary cortical neurons enter apoptosis following treatment with conditional media of rat HAPI (Highly Aggressively Proliferating Immortalized) microglial cells that have been exposed previously to TCDD. Exposure of microglial cells to TCDD triggers the rapid expression of inducible nitric oxide synthase and, subsequently, NO synthesis and p38/JNK activation [27]. A recent study showed that the AhR is expressed in mouse microglial cells and that it exerts both pro- and anti-inflammatory effects depending on the nature of AhR ligands (FICZ, TCDD, or 3-MC) [134]. These divergent responses may be the result of ligand-dependent processes. TCDD also influences the phenotype of astrocytes triggering a cytoplasmic Ca^2+^ increase, followed by the rapid expression of Src-suppressed-C kinase substrate (SSeCKS), and subsequently, protein kinase C- and TGF-β-activated kinase 1 (TAK1)-dependent NFkB signaling and TNF-α secretion [109,135]. This pro-inflammatory state might also favor neurotoxicity.

The toxicity of TCDD in the brain might depend on the genetic background of the organism, or the region of the brain that is being studied. In mice with a p53-deficient background, exposure to TCDD increases the amount of lipid peroxidation, DNA fragmentation, and cytochrome c reduction, probably through the generation of ROS [136]. The type of ROS produced in different regions of the brain is dissimilar, as shown by Hassoun et al., in rats. The cerebellum and the brain stem were not affected by exposure to TCDD [118].

## 6. Influence of AhR Ligands on Rodents’ Behavior and Neurotransmitter Levels

Effects having a relationship to behavior following exposure to AhR ligands have been described in the literature. Exposure of mice to TCDD (1 µg/kg) disrupts the circadian clock and decreases the expression of two “clock” genes, Per1 and Bma1 [29]. However, AhR knockout mice do not display any alteration of the circadian clock. This suggests that although it may have physiological functions, the AhR only becomes involved in the regulation of the circadian clock in order to adapt to environmental changes. Further, these changes may not only be related to exogenous signals. For example, when the rat SCN cell line (SCN2.2) is exposed to tryptophan metabolites (such as FICZ), there is an alteration in the expression of clock genes (including Per1), indicating that the activation of the AhR by exogenous or endogenous ligands may affect the circadian clock [137]. Per1 is also a target of TCDD in the hypothalamic CnV-3 cell line [49].

Several types of behavior are affected in mice exposed in utero and during lactation to low doses of TCDD (0.6 or 3.0 µg/kg). Mice exposed to the lower dose (0.6 µg/kg) display inflexibility, compulsive repetitive behavior, and lowered competitive dominance. Fewer behavioral alterations were observed following exposure to the higher dose. At the cellular level, hypoactivation of the medial prefrontal cortex and hyperactivation of the amygdala was demonstrated by immunohistochemistry (with the neuronal activation marker Arc). The results obtained with 0.6 or 3.0 µg/kg suggest that the effects of TCDD on mouse behavior may be non-monotonous [138]. However, another study using the same protocol with 3.0 µg/kg, described alterations of the contextual memory in the male mouse offspring [139]. Powers et al. found that littermates of AhR +/− mice exposed to TCDD (5 µg/kg, GD13) performed poorly as compared to untreated mice on a radial arm maze (to test spatial learning); in these mice, the field composed of the mossy fibers in the hippocampus was smaller than in the untreated mice [140].

The rat model has been used frequently to study the effects of exposure to TCDD on behaviors. Rat offspring exposed to TCDD (0.1 µg/kg/day GD9-19) displayed altered responses to motor, locomotor, and learning functions (motor development was delayed, longer latency in the active avoidance learning and decreased locomotor activity) [141]. An increase of the “brain volume/body weight” ratio was also observed in these animals. A study with human newborns conducted by the same group in parallel with the rat study described above showed that the concentration of TCDD in breast milk samples correlated inversely with head circumference [142]. The thickness of the cortex also was influenced by TCDD after gestational exposure, probably due to an alteration in the size patterns of cortical cells [143]. Moreover, a change in the dominance lateralization of the hemispheres was suggested, based on cell counting [143].

Some authors have looked for a link between altered behavior and stimulation of signaling pathways. For example, exposure to TCDD in utero altered the contextual fear conditioning of male rats. Contextual memory involves the hippocampus as a central coordinator, and phosphorylated CREB, which is a classical marker used to evaluate hippocampal activity, was found to be decreased in the CA1 region of these rats [144]. Previously, we described studies that showed that CREB is physically associated with the AhR on the promoters of target genes, and that NR stimulated CREB activity by phosphorylation. It is unknown at present for this study whether TCDD can affect the expression or the activity of NR, which can also affect AhR activity (NMDA inhibitors decrease expression of AhR target genes); however, one possibility would be that TCDD reduces this NR activity (see above in the cell survival), and subsequently the phosphorylation of CREB (and affects contextual memory). On the other side, the influence of the AhR on CREB stability or activity due to its physical association could also represent a mechanism that would need to be deciphered. Finally, exposure to a dioxin-like PCB (PCB126) of young rats inhibits the glutamate-NO-GMP cyclic pathway in the cerebellum, which is linked to deficits of learning ability; this is not observed with adult rats [145], but this is consistent with recent studies on zebrafish that show impaired habituation to novel environments in the case of developmental exposure to PCB126 (Glazer et al., 2016, Neurotoxicology).

TCDD also influences the levels of neurotransmitters and monoamines: the dopaminergic system is involved in the neurodevelopment and deregulation of this system leads to learning disability and hyperactivity disorder. Exposure of rats to TCDD (gavage, GD1 to lactation day 30) during gestation and lactation, increased the levels of monoamines, including dopamine, in the cerebellum [70]. Byers et al. observed an increase in the level of dopamine in the hypothalamus and in the brainstem of adult female Sprague-Dawley rats exposed to low levels of TCDD (46 ng/kg bw, daily, gavage) [146]. An increased level of dopamine is noted also in the midbrain of mice (TCDD 8 ng/kg, daily, gavage) [147]. This is coherent with the observation that TCDD increases the expression of tyrosine hydroxylase (TH), an enzyme that is involved in dopamine synthesis, and in the fetus, it is associated with an abnormal development of the midbrain dopaminergic system, a process that is potentially involved in the occurrence of autism-hyperactivity disorders (ADHD) [147,148]. Increased mRNA expression of TH triggered by TCDD is also observed in the neuroblastoma cell line, N2a-Rß [149].

Norepinephrine, serotonin, and GABA levels also are influenced by TCDD. Following the exposure of female rats to chronical low levels of TCDD (daily, gavage), the level of norepinephrine was increased in the hypothalamus, cerebral cortex, brainstem, and cerebellum, and there was an associated increase in the production of the superoxide anion (a ROS) [146]. Mice exposed in utero to TCDD (gavage) display a lower number of serotoninergic neurons in the brainstem (specifically in the raphe nuclei) [150]. Finally, in rats exposed to TCDD (gavage, GD1 to LD30) during gestation and lactation, there was respectively an increase in the levels of GABA in the cerebellum [70], and a decrease in the hypothalamus [51]. TCDD (10 nM) also upregulates the mRNA levels of GABA-R α2 and serotonin 2C-R (5HT2C) in the rat GnV-3 hypothalamic cell line [49]. The impact of TCDD or the AhR on the GABAergic system (see also *C. elegans*) could be useful for explaining several toxicities of TCDD. In utero exposure to TCDD causes feminization of adult male rats, which can be due to changes in the sexually dimorphic nucleus of the pre-optic area (SDN-POA). Whether TCDD causes a change in the volume of the SND-POA in the hypothalamus is controversial, since Ikeda et al. found a decrease in males rats exposed in utero (TCDD orally administered on GD15, 200 ng/kg, measure of the volume at PND98) [151] whereas Kakeyama et al. reported no changes with the same protocol [152]. However, the feminization also depends on the production of GABA in the SND-POA, in which the AhR is expressed in the same neurons as the glutamic acid decarboxylase 67 (GAD67, which produces GABA). There is a lower expression of GAD67 mRNA in males as compared to females. Further, there is a higher expression in females in most of the nuclei of the POA. However, in the antero-ventral periventricular nucleus (AVPV in the POA), exposure to TCDD (1 µg/kg body weight, GD15) eliminated this difference by reducing levels of GAD67 mRNA in the females. Following treatment with TCDD, the amount of GAD67 mRNA also was decreased in the medial pre-optic nucleus in males [153]. An effect of TCDD on GABAergic differentiation also has been described in other models: in mice embryos (E13.5) exposed to TCDD (gavage, GD 11.5), analysis of the telencephalon transcriptome suggested that decreased GABAergic differentiation was mediated by Sox11 down-regulation. Interestingly, decreased levels of Sox11 mRNA also are observed in AhR knockout (KO) mice [24]. Globally, exposure to TCDD in utero (1 μg/kg, gavage, GD15) mostly decreased the number of GABAergic neurons in several regions of the brain, including the medial prefrontal cortex (mPFC), the superior colliculus (SC), the amygdala, and the hippocampus [154]. Similarly, Powers et al. demonstrated that the number of mossy fibers in the hippocampus was decreased in AhR +/− mice exposed to TCDD (5 μg/kg, gavage, GD13) [140].

In addition to the effects on the levels of GAD67 and GABA, other mechanisms leading to the feminization of male rats might be affected by exposure to TCDD. During mating, Long-Evans male rats display an increased expression of BDNF mRNA in the frontal cortex, but, perinatal TCDD exposure (200 ng/kg, gavage, GD15) counteracts this effect [152]. Whereas feminization is not associated with alterations of the ER signaling pathways in dimorphic brain nucleus [155], the activity of aromatase, the estrogen-producing hormone, is decreased by treating rats at GD15 with 200 ng/kg of TCDD. As previously noted, the effects of TCDD may be non-monotonous, since treatment with 800 ng/kg has no effect.

In addition to neurons, other cell types in the brain could be targeted by AhR ligands, with subsequent actions on the development of pathologies and behavior. For example, TCDD affects the expression of myelination markers in oligodendrocytes of the rat brain; a single exposure during late gestation alters the mRNA levels of both PDGF-R alpha (a marker of oligodendrocyte differentiation) and MBP (a protein expressed in the myelin). PDGF-R alpha mRNA is increased in the diencephanlon at P2, and decreased in the cerebellum at P2 and P135. The level of MBP mRNA decreased in the cerebellum at P2 and P135, and in the telencephalon at P2 [74]. It is noteworthy that in a mouse model of demyelination (EAE, experimental autoimmune encephalomyelitis), TCDD at subchronic low doses counteracts the development of multiple sclerosis probably through suppression of interleukin 17-dependant processes [156]. This is also observed with dietary indole derivatives (indole-3-carbinol, diindoylmethane). In the EAE model, the positive curative effect of laquinimod (an oral drug which is currently tested to treat multiple sclerosis) probably targets the AhR [157]. Again, the context probably plays an important role on the effects of AhR ligands.

The toxicity of AhR ligands on the cells of the CNS might also result from indirect mechanisms such as the disruption of the blood brain barrier (BBB).

## 7. The AhR Regulates the Function of the Blood Brain Barrier (BBB)

As previously stated, the BBB plays a significant protective role against AhR ligands [116]. A very high expression of the AhR and the xenobiotics metabolizing enzymes (XME) battery was detected at the pig blood-brain interface, but also in the human cerebral microvascular endothelial cell line, hhCMEC/D3 [158,159]. Filbrandt et al. also detected the AhR in mouse endothelial cells from the BBB [25]. The authors also showed that treatment by well-known exogenous AhR ligands (such as BNF) increase the expression of XME, such as Cyp1a1 and Cyp1b1, as well as transporters including P-glycoprotein (Pgp) or multi-drug resistance-associated protein 2 (Mdr2). The results suggest that the AhR is functional in this endothelium. Dauchy et al. found that, in addition to being highly expressed in both cell types, the AhR is also functional since it can regulate the expression of CYP1A1 and 1B1 following exposure of the cells to TCDD (25 nM, 24 h) [160]. Overall, these studies have demonstrated a widespread expression of the AhR in brain endothelial cells, and they suggest that it regulates important detoxification functions.

Regarding the permeability of the BBB, a recent study in rats showed that TCDD accumulates primarily in the liver, with very little being found in the brain (0.01%). Similarly, only 0.02% of TCDD is transferred to the fetus if pregnant rats are exposed by gavage. However, the rate of transfer towards the fetal brain is 100 times higher in the fetus as compared to adults. This suggests that the fetal BBB is immature with respect to protection against pollutants, as compared to the placental barrier or the adult BBB [161]. The developmental stage is thus a very important factor when considering the potential toxicity of xenobiotics.

One mechanism that could explain a possible role for the AhR in the dysfunction of the BBB has been described by Chang et al. Using murine cerebrovascular endothelial cells, they demonstrated that 3-methylcholanthrene (3MC) triggered ß-catenin degradation (through the activation of the PKC/GSK-3ß pathway and ß-catenin phosphorylation). Consequently, decreases were observed in the expression of several genes such as fibronectin and α5ß1 integrin, which are implicated in adhesion between endothelial cells, and there was a reduction of the protein interactions at focal adhesion sites or adherens junctions [162]. A direct physical interaction between ß-catenin and the AhR has been demonstrated [163,164] and this interaction might play a role in maintaining the integrity of the BBB. Finally, perturbation of transporters (observed at very low doses, 50 pM of TCDD in rat brain capillaries) might have a significant impact on the accumulation or non-accumulation of co-contaminants thus aggravating the toxicity of mixtures.

Besides its role in the maintenance of the integrity of the BBB, the AhR may affect cerebral blood flow. In a zebrafish model, TCDD and the AhR2 increased the expression of Cyp1c1 and Cyp1c2 in the endothelium which resulted (as demonstrated by the use of morpholinos) in a decreased blood flow in the mesencephanlic vein [26]. This phenomenon has also been described in the midbrain. The same group previously demonstrated TCDD-induced cell death in the dorsal brain [104] and increased albumin (BSA) permeability of the barrier [165] following AhR2 activation. Moreover, the stimulation of cell death by TCDD in the dorsal midbrain appears to be dependent on the reduction of the local circulation [166,167]. Dong et al. showed that TCDD increased the levels of cyclooxygenase 2 (COX2), and that blockade of this induction protected the midbrain against apoptosis. The expression of thromboxane A synthase 1 (TBXS) is also increased, which suggests that the COX2-TBXS axis is involved in the reduction of blood flow and apoptosis following exposure to TCDD (probably through a non-genomic pathway) [168].

In addition to the use of toxic AhR ligands, AhR knockout models, developed during the last decade, have been of significant use to elucidate the functions of the aryl hydrocarbon receptor in the nervous system.

## 8. The Physiological Functions of the AhR in the Central Nervous System

The functions of several regions of the brain have been investigated in AhR KO animal models but to a far less extent than investigations that use TCDD as an AhR agonist. The expression of the AhR in neural progenitor cells in the mice hippocampus is consistent with the novel hypothesis that the AhR may play a role during cell differentiation, a hypothesis that is supported also by the results of investigations on mice embryos [24]. Latchney et al., while investigating the effects of AhR deletion and TCDD intoxication, observed that neuronal hippocampal differentiation was impaired, which resulted in defects of the contextual memory [20]. Dever et al., also demonstrated that disrupted expression of the AhR in the cerebellar granule neuron precursors impaired neurogenesis through the inhibition of precursor proliferation and an increase in differentiation [169].

The HPG axis is also affected in AhR KO mice. In particular, the expressions of lactotropin, prolactin-ß (Prl-ß) and the gonadotropins (GH and LHß) are reduced in the pituitary gland [18]. An inhibitory effect of ßNF (10–1000 nM, 20 h) on prolactin also was observed in the rat GH3 somatolactotrope cell line, which suggests that AhR ligands might act as a disruptor of AhR-regulated physiological functions (and similarly to AhR depletion) [18].

In the brainstem, the AhR has been implicated in the regulation of the cardiorespiratory system. The murine AhR positively regulates the expression of the Vav3 (a GTP-exchanging factor for Rac and Rho proteins) in the ventrolateral medulla, a region involved in the coordinated control of the cardiorespiratory system. Defects of the AhR or of Vav3 lead to the same phenotype in mice, which displays increased sympatho-excitation and decreased GABAergic transmission, which results in tachypnea [170].

We have demonstrated that AhR deletion in mice leads to the occurrence of a spontaneous pendular horizontal nystagmus. The mechanism involved in the production of the abnormal eye movements is probably a deficit of the visuo-motor circuitry, as suggested by a disrupted optokinetic reflex (OKR) [23]. The visuo-motor circuitry is a complex structure composed of several parts, and further investigation is necessary to elucidate the role of each of the parts. However, one of the mechanisms involved in this nystagmus, might be the disruption of the integrity of myelin. Indeed, we observed an altered optic nerve myelin sheath that was associated with the down-regulation of myelin-associated glycoprotein (MAG), one of the key proteins involved in myelination by the oligodendrocytes. Local inflammation is also observed, which contributes to the down-regulation of MAG [171]. Beside the central nervous system, the myelin structure of the peripheral nervous system is also affected, displaying similar disrupting patterns [172]. The suspected mechanism involved in the down-regulated expression of myelin proteins, would be an over-activated ß-catenin pathway, as absence of the AhR does not counteract the transactivating function of ß-catenin, which binds and inhibits the promoter activity of myelin genes.

In the visual system, focus on the retina has been undertaken in AhR KO mice. The AhR has been implicated in the occurrence of the neovascular subtype of age-related-macular degeneration (AMD) with dysfunction of the retinal pigment epithelium (RPE, a cell subtype which also expressed the AhR in humans [173]) leading to local inflammation, increased secretion of collagen IV, and coherently, increased levels of the pro-fibrogenic TGF-ß1 [174]. Kim SY and colleagues also observed an atrophy of these cells, together with a sub-retinal accumulation of microglial cells [175]. In wild-type mice, the use of a synthetic AhR ligand protects the degeneration of RPE by alteration of lipid metabolism [176]. As stated before, the AhR is also expressed in the RGC of the mouse retina, and the dysregulation of such cells in AhR KO mice could be also a key event in the occurrence of the spontaneous pendular horizontal nystagmus. This is reminiscent of experiments using TCDD as an exposure stress. Indeed, in the swin-up rainbow trout, exposure to TCDD decreased the densities of the RGC, which led the authors to suggest that visual deficits and less efficient prey capture is associated with TCDD exposure [177]. Interestingly, other members of the AhR battery genes are involved in retinal function, as deletion of the AhRR in the zebrafish model alters the expression of genes involved in photoreceptor function [178].

Williams et al. also demonstrated that the constitutive activity of the AhR is important for the spontaneous movement in mice, as demonstrated by measurements of the activities of 43 different strains [179].

A recent study also suggests that AhR deletion in mice leads to adaptive mechanisms such as increased kynurenic acid levels (due to higher expression of the producing enzyme kynurenine (KYN) aminotransferase II), which is described as an endogenous ligand of the AhR and more importantly, as an antagonist of NMDA receptors, promoting a decreased excitotoxicity of glutamate [180]. This is coherent with genome-wide association studies (GWAS) that show an association between AhR expression (and the regulation of KYN-biosynthesizing enzymes, together with the levels of KYN) and the severity of major depressive disorders [181], or by a recent study on the damages induced by the KYN/AhR following experimental stroke [182]. One suspected mechanism would be similar to the demyelinating processes observed in AhR-knockout mouse, a stimulated recruitment of pro-inflammatory monocytes and activation of astrocytes by chemokine ligand 2 (CCL2) whose expression would be enhanced by KYN [183].

## 9. Conclusions

The AhR is expressed in several neural cell types including neurons, astrocytes or microglial cells. Its functions in the nervous system has been mainly studied using TCDD which is a well-known persistent organic pollutant but also a non-genotoxic xenobiotic. This molecule exerts its toxicities mainly through binding and activation of the AhR, with caution that need to be taken regarding the time course of the experiments, the developmental stage of the model used, and the possibility of a biphasic effect of such a molecule (see the effect on orexigenic factors). Besides xenobiotic metabolism enzymes, several target genes expressed in different parts of the brain have been identified; their regulation may help to explain the TCDD toxicities. Xenobiotic AhR ligands could also exert their toxicities by competitive binding on the AhR with endogenous ligands, including tryptophan metabolites such as kynurenin. Studies from our laboratory have shown that different AhR ligands do not lead to the same transcriptional responses; according to the nature of its ligand, the AhR may bind different responsive elements which differ from one or two bases [39]. Therefore, we hypothesize that xenobiotic AhR ligands could act as disruptors, and this is partly confirmed by similar phenotypes observed in TCDD-exposed animals and AhR knockout animals. For example, neuronal hippocampal differentiation is impaired in both models, resulting in defects of the contextual memory [20]. Another example is the influence of the AhR on the differentiation of GABAergic neurons (and Sox11 down-regulation). Dysfunction of hippocampal GABAergic interneurons have been recently associated with schizophrenia; interestingly, a post-mortem proteomic study on human schizophrenic patients identified AhR signaling as one disrupted function of their hippocampus [184].

The regulation of the differentiation of GABAergic neurons also revealed that the effect of AhR signaling is sex-dependent, an effect that is concordant with a sex-dependent alteration of reproductive functions observed with several animal models exposed to low doses of TCDD. For example, demasculinization of sexual behaviors is observed with male Holtzman and Long Evans rats perinatally-exposed to TCDD (while interfered defeminization is not necessarily observed) [185,186]. These observations highlight the need of experiments that use both sexes in order to identify any sexual dimorphic effect involving the AhR signaling.

Those observations raise the questions of the roles of endogenous ligands such as kynurenin, but recent studies have also characterized AhR ligands in the food (cruciferous vegetables, CV) or in the microbiota (as virulence factors). It is then tempting to ask what the influence of this food composition is (tryptophan-rich or CV-rich diets) on the brain functions regulated by the AhR. Similarly, *Caenorhabditis elegans* might eat soil bacteria containing AhR ligands, which might explain why AhR-1 is so important for feeding behavior [7]. In vertebrates, the AhR is expressed in the hypothalamus, which is also a central mediator for feeding behavior.

The AhR might then be reconsidered as an environment sensor, and it is interesting to note that it is highly expressed in sensory parts of the nervous system in vertebrates such as the eye or the olfactory bulb.
